# Redox-Active Metal Ions and Amyloid-Degrading Enzymes in Alzheimer’s Disease

**DOI:** 10.3390/ijms22147697

**Published:** 2021-07-19

**Authors:** Namdoo Kim, Hyuck Jin Lee

**Affiliations:** 1 Department of Chemistry, Kongju National University, Gongju 32588, Chungcheongnam-do, Korea; ndkim123@kongju.ac.kr; 2 Department of Chemistry Education, Kongju National University, Gongju 32588, Chungcheongnam-do, Korea

**Keywords:** redox-active metal ions, Cu(I/II), Fe(II/III), metal chelators, amyloid-degrading enzymes, neprilysin, insulin-degrading enzyme, ADAM10

## Abstract

Redox-active metal ions, Cu(I/II) and Fe(II/III), are essential biological molecules for the normal functioning of the brain, including oxidative metabolism, synaptic plasticity, myelination, and generation of neurotransmitters. Dyshomeostasis of these redox-active metal ions in the brain could cause Alzheimer’s disease (AD). Thus, regulating the levels of Cu(I/II) and Fe(II/III) is necessary for normal brain function. To control the amounts of metal ions in the brain and understand the involvement of Cu(I/II) and Fe(II/III) in the pathogenesis of AD, many chemical agents have been developed. In addition, since toxic aggregates of amyloid-β (Aβ) have been proposed as one of the major causes of the disease, the mechanism of clearing Aβ is also required to be investigated to reveal the etiology of AD clearly. Multiple metalloenzymes (e.g., neprilysin, insulin-degrading enzyme, and ADAM10) have been reported to have an important role in the degradation of Aβ in the brain. These amyloid degrading enzymes (ADE) could interact with redox-active metal ions and affect the pathogenesis of AD. In this review, we introduce and summarize the roles, distributions, and transportations of Cu(I/II) and Fe(II/III), along with previously invented chelators, and the structures and functions of ADE in the brain, as well as their interrelationships.

## 1. Introduction

In the human brain, various metal ions are essential as cofactors for numerous enzymes for catalytic activities and neurotransmission including synaptic plasticity, myelination, and synthesis of neurotransmitters [1,2]. Moreover, redox-active metal ions [i.e., Cu(I/II) and Fe(II/III)] have critical roles in oxidative metabolism. Therefore, the homeostasis of metal ions is tightly regulated [1,2,3,4,5,6]. Dyshomeostasis of Cu(I/II) and Fe(II/III) could overproduce reactive oxygen species (ROS) via Fenton-like reactions to elevate oxidative stress and induce the malfunctioning of mitochondria. Additionally, defects in energy metabolism, aberrant axonal transport, and inflammation have been observed which potentially lead to neurodegenerative disorders [7,8,9,10]. Once the redox-active metal ions bind to amyloid-β (Aβ), a major risk factor of Alzheimer’s (AD), rapid peptide aggregation and formation of toxic oligomeric species are observed, along with overproduction of ROS [11,12,13,14]. Therefore, the redox-active metal ions, Cu(I/II) and Fe(II/III), could be related to neuronal impairments, subsequently leading to cognitive defects. In order to reduce the risk of neurodegeneration by Cu(I/II) and/or Fe(II/III), the metal chelation strategy has been suggested as the treatment of AD; however, only targeting metal ions could not cure the disease completely (vide infra).

In addition to redox-active metal ions, clearance of Aβ in the brain is critical for ameliorating neurotoxicity. Amyloid degrading enzymes (ADE), including neprilysin (NEP), insulin-degrading enzyme (IDE), and ADAM10, are involved in the Aβ removal process to regulate the protein levels in the brain [15,16,17]. Since the levels and activity of ADE have been reported to be decreased with aging, the risk of AD occurring increases [15,18]. Thus, enhancing the levels and/or actions of ADE could be a potent therapeutic strategy to AD [18,19]. Moreover, although the redox-active metal ions could affect the activity and levels of ADE, the influence of Cu(I/II) and Fe(II/III) on the enzymatic activity of ADE still needs to be investigated in many aspects.

In this review, we summarize the distributions and roles of redox-active metal ions [i.e., Cu(I/II) and Fe(II/III)] in both healthy and diseased brains, as well as previously developed chemicals to regulate the levels of those metal ions. Moreover, the effect of Cu(I/II) and Fe(II/III) on the activity and/or levels of ADE is also introduced.

## 2. Cu(I/II)

### 2.1. Cu(I/II) Distributions in the Nervous System

The Cu ion is the third most abundant transition metal ion (ca. 100 mg) in the human body [20]. It works as a cofactor that binds to various metalloenzymes and assists their activation [4,21]. Since it usually exists as cuprous ions [Cu(I)] and cupric ions [Cu(II)], Cu(I/II) can serve as an electron transporter. Cu(I) has an electron configuration of [Ar]3d^10^, and Cu(II) has [Ar]3d^9^ [20,22]. In addition, Cu(I/II) plays a key role in energy metabolism, signal transduction, reproduction, and development that are very important for physiological functions. For example, cytochrome c oxidase in mitochondria needs Cu ions for its activation, and dopamine-β-hydroxylase utilizes it for the cellular secretory pathway [23,24,25,26]. Cu ions can be detected in various points of the brain such as the soma of cortical pyramidal and cerebellar granular neurons, neuropil within the cerebral cortex, hippocampus, cerebellum, and spinal cord [27]. On average, ca. 100 μM of Cu ions has been detected in the brain; however, some parts of the brain have a 2- to 3-fold higher concentration than the other regions [28]. In particular, the ceruleus has a 1.3 mM concentration, which is a part of the brain related to stress and panic. The substania nigra, the dopamine-producing region of the brain, also has a high Cu ion concentration (ca. 400 μM) [28].

The extracellular level of Cu ions depends on the cellular environment. In cerebrospinal fluid (CSF), only 0.5–2.5 μM of Cu ions exists, while the synaptic cleft contains 30 μM [29,30,31]. Usually, the Cu ion concentration is 2–3-fold higher in neurons [32]. In the brain, Cu ions exist in two types: (i) those tightly bound to the proteins, or (ii) those in labile pools [33]. Several regions of the brain such as the soma of cortical pyramidal and cerebellar granular neurons, the hippocampus, the cerebellum, and the spinal cord have labile copper stores [27]. There are also labile pools with a low concentration in the extracellular regions [34].

### 2.2. Regulations of Cu by Metallochaperones

#### 2.2.1. Copper Chaperone for Superoxide Dismutase (CCS)

A 54 kDa metalloprotein, CCS, is found in the cytosol, mitochondria, and nucleus and transports Cu(I) to superoxide dismutase (SOD1) which is the major antioxidant [35,36,37]. For its activation, SOD1 needs catalytic metal ions such as Cu or Mn ions. CCS has three domains for its function: (i) domain I at the N-terminus with Cu binding motif MXCXXC, (ii) domain II with a similar structure to SOD1 which can bind to SOD1, and (iii) domain III containing Cu(I) with the CXC Cu binding motif [35,36,38]. Once docked to SOD1, a disulfide bond is formed between Cys244 of CCS and Cys57 of SOD1, leading to activation of SOD1. On the other hand, SOD1 activity was greatly decreased (70–90%) upon the *CCS* gene [35,36].

#### 2.2.2. Antioxidant Protein 1 (Atox1)

Atox1 is a cytosolic metallochaperone protein that delivers cytosolic Cu to ATP7A and ATP7B via a ligand exchange [39,40,41]. The role of Atox1 is to protect cells from attacking ROS. Sequestrated Cu ions by Atox1 are transferred to the trans-Golgi network (TGN) of secretory vesicles [42,43]. Atox1 consists of four β-sheets and two α-helices forming a βαββαβ structure. First βα has a Cu binding motif, MXCXXC, and binding of Cu(I) induces a conformational change to form a bent S–Cu(I)–S bond. Recently, it has been found that tumors need a high level of Cu ions. Breast cancer cells need Atox1 for migration and identification of partner proteins [44].

#### 2.2.3. Cytochrome C Oxidase Assembly: Cox11, Cox17, Cox19, Sco1, and Sco2

Cox11, a 28 kDa metallochaperone, is located in the mitochondrial inner membrane. A single transmembrane helix allows it to be tethered to the membrane [45]. The Cu(I) binding domain is located at the C-terminus of the protein, which forms a dimer. Each monomer can coordinate one Cu(I) via three thiolate ligands [46]. It has been reported that Cox11 helps the formation of the Cu_B_ site of cytochrome c oxidase [47]. On the other hand, Cox17, an 8 kDa metallochaperone, delivers Cu(I) to form both Cu_A_ and Cu_B_ sites [48,49]. Cox17 is found in the cytosol and mitochondrial intermembrane space. Among the seven Cys residues of the protein, three Cys residues are important for the function of Cox17 (CCXC motif), and one Cox17 can bind to three Cu(I) [50]. This protein delivers Cu(I) to Sco1, an intermediate protein on the way to cytochrome oxidase [51]. Another Cu(I)-binding protein, Cox19, has a similar structure to Cox17, and it is located in the mitochondrial intermembrane space as well. Cox19 is expected to contribute to the transportation of Cu ions into cytochrome c oxidase [52,53].

Both Sco1 and Sco2 are cytochrome c oxidase assembly proteins which are Cu metallochaperones [54,55,56]. Sco1 is a 33.8 kDa protein with three Cu binding amino acid residues at Cys169, Cys173, and His260. It has 7 α-helices, 10 β-strands, and 2 turns [57,58]. Its location is in the inner mitochondrial membrane, where it transports Cu ions to the Cu_A_ site on Cox2. The other important role is controlling the localization and abundance of Ctr1 for Cu homeostasis [59,60]. Cytochrome c oxidase assembly becomes defective when there are mutations in Sco1 and Sco2, inducing Cu ion deficiency [61]. Sco2 is a 15.1 kDa protein consisting of 136 amino acids and is important for transferring Cu ions to the Cu_A_ site of cytochrome c oxidase subunit II. The redox state of the Cys residue in Sco1 is also regulated by Sco2, which is a thiol-disulfide oxidoreductase [61,62].

### 2.3. Uptake of Cu(I/II) through Blood–Brain Barrier

Important regions in the brain for regulating the uptake and release of Cu(I/II) are the blood–brain barrier (BBB) between the blood and brain interstitial fluid, and the blood–CSF barrier (BCB) between the blood and CSF [63,64,65]. The intracellular concentration of Cu ions should be tightly controlled since they can produce harmful chemical species (e.g., ROS) during the oxidation/reduction process [64,66,67]. Copper transporter-1 (Ctr1), ATP7A, ATP7B, glutathione (GSH), metallothioneins (MTs), and Cu chaperone regulate Cu ion transportation [68]. Reduction of Cu(II) to Cu(I) should be required before entering the brain [69,70]. In yeast, Fre1p and Fre2p, ferric reductases, are responsible for this process [69,70,71]. Reduced Cu(I) can be transported into the brain via multiple pathways driven by numerous proteins [72,73,74]. A more detailed description of this process, along with the related proteins, is summarized in the following sections.

#### 2.3.1. Copper Transporter-1 (Ctr1), Ctr2, and Ctr6

Ctr1 was first discovered in *Saccharomyces cerevisiae* as a high-affinity Cu uptake protein. It is a membrane protein composed of 190 amino acids and that is involved in the transport of Cu ions from the blood to cells [72,73]. The N-terminus is an extracellular part which is less conserved, while the C-terminus is a cytoplasmic part and highly conserved. Between them, there are three transmembrane domains. Met- and His-rich motifs (^7^MGMSYM^12^, ^40^MMMMPM^45^, ^3^HSHH^6^, and ^22^HHH^24^) at the N-terminus are involved in Cu binding [74,75,76]. Fluorescence resonance energy transfer (FRET) experiments revealed that Cu binding to the extracellular part of Ctr1 induces a conformational change in the cytosolic part, and this conformational change is a driving force for releasing Cu ions into the cytosol [77,78,79]. Ctr1 has a *K*_m_ of 2–5 μM and does not need ATP consumption for Cu ion transport. Ctr1 only uptakes Cu(I), not Cu(II), and Ctr1 transports Cu ions in both the liver and intestine of mice, allowing various proteins to function in the cytosol [80]. Ctr1 is mainly present in the plasma membrane and the intracellular vesicle. Under a high Cu concentration, Ctr1 moves from the plasma membrane to the cytosol and is decomposed [74]. The *Ctr2* gene found in the human genome has homology with Ctr1, but it differs from Ctr1 in that there is no extended portion of the N-terminus, but the sequences of the transmembrane domain, which are considered essential for Cu transport, are almost the same [81,82]. Unlike Ctr1, the proportion of Ctr2 distributed on the plasma membrane is less than 5%, and the rest is intracellular [83,84]. Therefore, it was expected to have a different function from Ctr1, suggesting that the absence of the N-terminus of Ctr2 would function as Cu uptake in the plasma membrane with a low affinity. Recent studies revealed that the vacuole can store and mobilize Cu ions [85,86]. Ctr6, a newly discovered Cu transporter in *Schizosaccharomyces pombe*, is translated under Cu-deficient conditions. Both Ctr2 and Ctr6 are localized to the vacuole, and their functions transfer Cu ions stored in vacuoles to the cytosol [87,88].

#### 2.3.2. ATP7A and ATP7B

ATP7A and ATP7B are homologous ATP-driven transporters, types of ATPase. Their size is 160–170 kDa, containing eight transmembrane domains and several cytosolic domains. The N-terminus is located in the cytosol and has six Cu binding motifs consisting of GMXCXXC [89]. ATP hydrolysis occurs at the ATP binding domain located between transmembrane domain 6 (TM6) and TM7. The A-domain between TM4 and TM5 plays a role in inducing a conformational change during ATP hydrolysis [90,91,92]. Normally, they are located in the TGN and receive Cu ions from the Cu chaperone Atox1 into the Cu binding domain. This causes a conformational change, and Atox1 can deliver different Cu ions to different binding sites [93,94]. Cu ions are transferred to the TM domain by ATP hydrolysis and phosphorylation of Asp residues, which cause Cu ions to be exposed to the lumen of TGN. ATPase is dephosphorylated while releasing Cu ions and returns to its original state. The low pH of TGN plays a key role during this action [95]. There is about 60% sequence homology between ATP7A and ATP7B, but the functional aspects are not identical. ATP7A is faster, whereas ATP7B has a higher affinity for Cu [96,97]. Unlike Ctr, they are responsible for the transport of Cu ions out of the cytosol using the energy from ATP hydrolysis. The released Cu ions move to the other tissues by ATP7A and to the bile by ATP7B. Since neither ATPase exists in the plasma membrane, Cu ions are not directly transported across the plasma membrane but are transported to intracellular vesicles, allowing the vesicles to be fused with the plasma membrane for release [98,99,100]. Additionally, these Cu-APTases deliver Cu ions to many Cu-binding proteins. Cu-binding proteins in the plasma membrane receive Cu ions while ATP7A and APT7B export Cu ions through the vesicle [101].

#### 2.3.3. Glutathione (GSH)

GSH is a tripeptide (Glu-Cys-Gly) existing in high concentrations (0.5–10 mM) which is deeply involved in the oxidation/reduction reactions occurring in the body. As an antioxidant, it prevents cell damage caused by ROS and heavy metals [102,103]. There are a reduced form of GSH and an oxidized form of GSSG, and the ratio between these two forms is a measure of the oxidative stress of cells [104]. In normal cells and tissues, the GSH form is more than 90% [105]. Cu(II) is reduced by GSH to become Cu(I), and then it forms a Cu(I)–GSH complex [106]. GSH plays a role in regulating the function of Cu ion uptake by Ctr1 [107,108]. It is known that GSH also regulates the functions of ATP7A and ATP7B, which are involved in the transport of Cu ions, by regulating the binding of Cu ions to ATPase [109,110].

#### 2.3.4. Metallothioneins (MTs)

MTs are small molecule proteins present in almost all living organisms and have very high affinity with essential or toxic mono- and divalent transition metal cations. Due to their Cys-rich characteristic, they form metal–thiolate clusters selectively with metal ions having an electron configuration of d^10^ [111]. In particular, the binding to Zn(II) or Cu(I) plays a biologically important role [112,113,114]. They protect the body by releasing highly toxic transition metal ions and reduce oxidative stress induced by ROS and/or reactive nitrogen species [112,115]. There are four types of mammalian copper MT, from 1 to 4, and they consist of 61 to 68 amino acids. A total of 20 Cys are arranged in the form of Cys-Cys, Cys-X-Cys, and Cys-X-X-Cys [116,117]. They have a shape similar to a dumbbell, and two metal-thiolate clusters at both ends are connected by flexible sequences. MTs also exist in the liver, kidneys, and intestines and are widely distributed in the brain and neurons [118,119].

### 2.4. Cu in Normal and Diseased Conditions

#### 2.4.1. Cu in Nervous Systems under Normal Conditions

**Figure 1:**
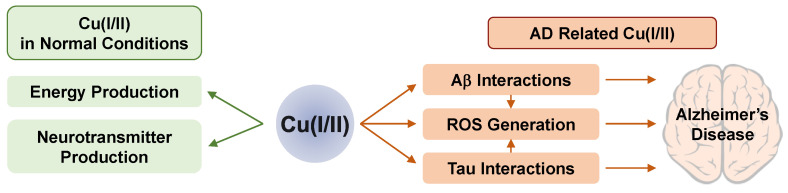
The roles and influence of Cu(I/II) on normal (left) and AD-affected (right) conditions.

In normal conditions, Cu ions have two important physiological roles. First, Cu(I/II) provides a driving force for several oxidation/reduction reactions performed by enzymes. As a cofactor for various enzymes, Cu(I/II) becomes a redox-active metal center in those enzymes [120]. In an electron transport chain occurring in mitochondria, Cu(I/II) can regulate the levels and activity of enzymes for energy production. In addition, Cu(I/II) regulates neurotransmitters, neuropeptides, and dietary amines. Second, Cu(I/II) acts as a signal and excitotoxicity modulator in neurotransmission [120]. In the brain, specific enzymes such as dopamine β-monooxygenase (DβM), peptidylglycine α-hydroxylating monooxygenase (PHM), tyrosinase, and Cu amine oxidase use Cu ions for their cofactor [121]. Labile Cu ions are also utilized for neuronal activity in the brain. Upon depolarization, micromolar range of Cu ions is excreted to the extracellular space using hypothalamus tissues or synaptosomes [34,122]. The intracellular origin of these Cu ions is ATP7A activated by NMDA receptors [123]. The basic roles of Cu(I/II) in the normal brain are summarized in Figure 1.

#### 2.4.2. Cu under Diseased Conditions

It is important to maintain the homeostasis and precise compartmentalization of metal ions in the neuronal signaling process to prevent toxic effects and dysfunctional activity [1]. Failure in homeostasis and compartmentalization can induce neuronal toxicity, eventually leading to AD. Moreover, redox-active Cu(I/II) binds to Aβ, which can generate ROS by Fenton chemistry and/or the Haber-Weiss reaction through the redox cycle, leading to oxidative stress, and form toxic Cu–Aβ aggregates which could lead to neurotoxicity and neurodegeneration [4,20,22]. About 0.4 mM of Cu ions was present in the senile plaques composed of Aβ aggregates in the brain of AD patients [4]. The dissociation constant for Cu(II)–Aβ is in the nanomolar range, which is found under a physiological environment [124,125]. There are two components of Cu(II) binding to Aβ depending on the pH; at a physiological environment, Aβ favors Cu(II) binding via component I, while under a basic environment, Aβ prefers component II [126,127]. In the synaptic cleft, Cu ions play an important role as a secondary messenger, and the concentration of Cu ions is estimated as ca. 100 to 250 μM, depending on the size of the cleft [128]. In addition, it was observed that the degree of Cu ion excretion into the extracellular space increased through depolarization by 50 mM of K(I) [34]. In a study about the binding of Cu ions to Aβ at the synaptic cleft using reaction-diffusion simulation, the binding of Zn(II) was very low, about 0.1% of the entire Aβ, while the binding of Cu ions was high enough to reach 30%. Therefore, Zn(II) does not significantly affect the formation of Aβ dimers in the neurotransmission process at the synaptic cleft, but it is believed that Cu ions play an important role in the early stages of Aβ oligomerization [129].

Cu–Aβ acts as a catalyst in oxidizing monoamine-based neurotransmitters such as dopamine, epinephrine, and serotonin. In particular, dopamine has an 85-fold higher tendency than autoxidation in Cu–Aβ_40_ [130]. Among the various Cu–Aβs, Cu–Aβ_16_ showed the greatest activity in the oxidation of several monoamine-based neurotransmitters. In the presence of hydrogen peroxide, ROS were generated in pathological conditions, and the catalytic capacity of Cu–Aβ was observed to increase, and it is believed that this may affect AD by promoting the oxidation of neurotransmitters. Conversely, it was known that direct interaction between Cu–Aβ and dopamine/dopamine derivatives can inhibit the aggregation of Cu–Aβ [131]. According to Nam et al., dopamine and its derivatives promote the oxidation of Aβ_40_ and Aβ_42_ in the presence of Cu(II), leading to modifying the aggregate form of Aβ. Instead of a general fibril form, amorphous and more compact forms were generated, or they decomposed existing Aβ aggregates [131].

In the case of Aβ found in the brain of AD patients, there are many N-terminally truncated forms, especially Aβ_4-x_. This form contains an H_2_N-X-X-His (ATCUN) motif that has a higher Cu(II) affinity than Aβ_1-x_ [132]. Glutamate, one of the neurotransmitters, promotes the transfer of Cu(II) from Cu–Aβ_4-16_ to Zn7-metallothionein-3 (Zn7-MT-3). This indicates that glutamate, which is instantaneously elevated during neurotransmission, forms an instant ternary complex with Cu–Aβ_4-16_ and promotes the transmission of Cu(II) [133].

In addition to Aβ, Cu ions can also interact with the tau protein which might be a potential pathogen for AD. Depending on the pH and stoichiometry, the R2 and R3 domains of tau can be Cu ion binding sites [134]. The His residue in this region is sensitive to pH changes, and it is responsible for Cu ion binding. Since R3 has two His residues close to each other, its binding affinity to Cu ions is higher than that of R2. The His residue in the R1 region can also bind to Cu(II), but its affinity is much weaker than the others [135,136]. The simplified effects of Cu(I/II) in the AD-affected brain are presented in Figure 1.

### 2.5. Regulators of Cu(I/II)

**Figure 2:**
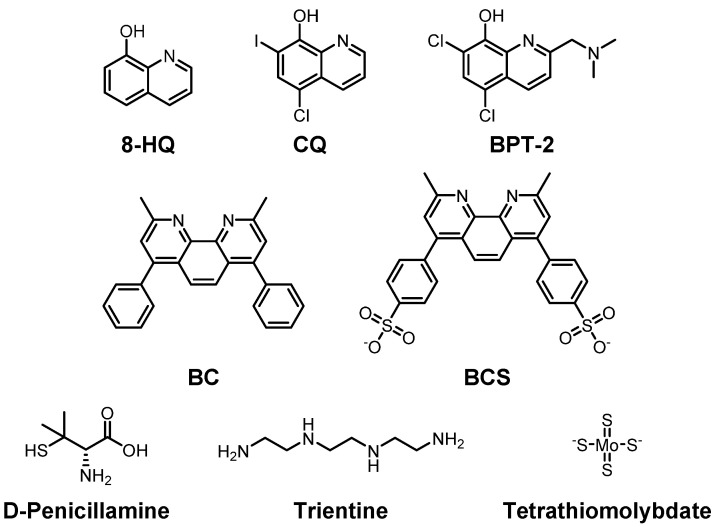
Selected Cu(I/II)-targeting molecules developed to maintain Cu(I/II) homeostasis. Top row: 8-hydroxyquinoline (**8-HQ**) and its derivatives, **clioquinol** (**CQ**), 5-chloro-7-iodoquinolin-8-ol, and **BPT-2**, 5,7-dichloro-2-[(dimethylamino)methyl]quinolin-8-ol; middle row: **bathocuproine** (**BC**), 2,9-dimethyl-4,7-diphenyl-1,10-phenanthroline, and **bathocuproine disulfonate** (**BCS**), 4,40-(2,9-dimethyl-1,10-phenanthroline-4,7-diyl)dibenzenesulfonate; bottom row: **D-penicillamine**, (2S)-2-amino-3-methyl-3-sulfanylbutanoic acid, **trientine**, triethylenetetramine, and **tetramolybdate**.

To reduce the risk of onset and/or progression of AD by Cu(I/II), various chemical agents have been developed to regulate the levels of the metal ions (Figure 2). The chemicals were examined by their ability to bind to Cu(I/II) in numerous studies. In this section, we summarize the chemical agents capable of interacting with Cu(I/II).

**Clioquinol** (**CQ**), an 8-hydroxyquinolin (**8-HQ**) derivative, is a candidate for treatment of AD by targeting Cu(II) and Aβ (Figure 2; top row) [137,138,139]. The **CQ**–Cu(II) complex has a square planar structure where two **CQ**s coordinate Cu(II) in a crystal structure. In the solution phase, its symmetry is broken, and it becomes a tetragonally distorted structure [140]. Regardless of this, the structure in solution is still closely related to that observed in crystallography. It has nanomolar affinity for Cu(II) and can cross the BBB [141]. In 2003, Prana Biotechnology reported the results of a phase II trial on **CQ** in AD patients due to its abilities of metal chelation and modulation of metal-free and metal-bound Aβ aggregation. However, further processes were stopped during the phase II/III trial since a toxic compound was detected during the manufacturing steps. Although it showed toxicity, **CQ** has recently been examined for its potential neuroprotective effect in *C. elegans* [142].

**PBT-2** is also an **8-HQ** derivative and the next generation of **CQ** developed for treatment of AD and Huntington’s disease (Figure 2; top row) [143,144]. It has improved BBB penetrance and pharmacokinetics compared to **CQ** [145]. Interactions between Cu ions and Aβ are disrupted by **PBT-2**, preventing the accumulation of toxic Aβ species in the brain. The extracellular Cu ion concentration is reduced since **PBT-2** transports Cu ions to the intracellular space. This effectively reduces the chance for Cu–Aβ interactions which could trigger Aβ aggregation [146,147]. Prana started the first phase II trial of **PBT-2** for AD in 2007, and the second phase II trial in 2011. In 2014, Prana reported that there is not much difference between treated and non-treated groups [148]. An additional phase II trial revealed that 250 mg/day, which the patients could tolerate, of **PBT-2** has positive results in terms of cognitive ability [149,150]. The clinical trial information of **PBT-2** is summarized in Table 1.

**DP-109** and **DP-460** are lipophilic chelators that chelate Cu, Fe, and Zn in the membrane [151]. In AD mice, **DP-109** administration reduced amyloid plaques and the degree of cerebral amyloid angiopathy in the brain [152]. Furthermore, both molecules slightly extended (10% and 9%, for **DP-109** and **DP-460**, respectively) the life span of G93A-transgenic ALS mice [153]. However, a recent study presented that **DP-460** has detrimental effects on learning based on the Morris water maze test [154]. Moreover, **bathocuproine** (**BC**) and **bathocuproine disulfonate** (**BCS**), presented in the middle row of Figure 2, could chelate Cu(I) and Cu(II) by N as electron donor atoms near methyl groups. **BC** prefers Cu(I) to Zn(II) due to its size [155]. **BCS** has two negative charges which provide water solubility to the molecule. Thus, **BCS** can be used to chelate extracellular Cu(II) as well [156,157,158].

Multiple chelators against Cu have been developed to treat other diseases such as Wilson’s disease. **D-penicillamine** is one of those Cu ion chelators (Figure 2; bottom row) [159,160,161]. It chelates and reduces Cu(II), which is then excreted in the urine at up to 1.5 mg/day, which is a four-five times increased amount compared to untreated cases in patients [162]. Additionally, upon treatment of **D-penicillamine** in a hydrogel form, it could improve the cognitive ability of APP/PS1 mice through the activation of ADAM10 [163]. **Trientine** (triethylenetetramine) is a selective Cu(II) chelator that suppresses oxidative stress. Once Cu(II) binds to **trientine**, a Cu(II)–**trientine** complex is excreted in the urine, but not as much as **D-penicillamine** (Figure 2; bottom row) [164,165].

**Tetrathiomolybdate**, shown in the bottom row of Figure 2, consists of a central molybdenum surrounded by four sulfhydryl groups and has been suggested as a therapeutic agent for Wilson’s disease to control the levels of Cu(I/II) as well. Once it binds to Cu ions in foods, it forms a very stable complex and is excreted in the stool [166]. **Tetrathiomolybdate** also forms a complex with serum albumin and labile Cu ions in the blood. Since Cu–**tetrathiomolybdate** cannot be reabsorbed, the complex is metabolized in the liver. The resulting metabolized fragments are excreted in bile. Moreover, upon treatment with **tetrathiomolybdate**, the production of inflammatory cytokines decreased in APP/PS1 transgenic mice [167]. If **tetrathiomolybdate** is used at more than a specified dose of 120 mg/day, two side effects might appear, namely, anemia, and elevation of the transaminase level [168].

There are several multifunctional molecules where other scaffolds are conjugated to metal-chelating moieties for reducing possible side effects or improving metal chelation for treatment of AD [1]. Since there are many factors that cause AD, multifunctional chemical agents that target multiple risk factors of the disease would be an effective strategy to treat/cure AD. In particular, many chemical agents targeting both Cu(I/II) and Aβ or Cu(I/II) and ROS have been developed. Based on multifunctionality, a few chemical agents, although they have firstly been suggested as Cu(I/II) chelators, are still examined for their potentials as therapeutic agents for AD in various animals (Table 2) [142,152,153,154,163,167].

More than ten multifunctional molecules based on **8-HQ** have been developed. They have tetra-O-benzyl-β-D-glucopyranoside, rasagiline, trehalose, glutathione, and β-cyclodextrine conjugated to **8-HQ** [169,170,171,172,173,174,175,176]. By applying **thioflavin-T** (**ThT**), an imaging agent for aggregated Aβ, several multifunctional molecules were developed by conjugating **CQ**, **DTPA**, **di-(2-picolyl)amine**, and/or ***N*-(2-pyridylmethyl)amine** [177,178,179,180,181,182,183,184]. Multifunctional molecules based on ***p*-I stilbene** (**pISTIB**) can chelate Cu(II) to regulate Cu(II)-induced Aβ aggregation [185,186,187,188,189,190,191,192,193,194]. Even though the exact role of triazole has not yet been elucidated, triazole-based chemicals have been developed having a quinoline ring and a phenol [195,196,197]. Multifunctional molecules affected metal-free Aβ aggregation, and others reduced Cu(II)-induced Aβ aggregation when **selegiline**, **aurone**, and **chromone** were conjugated [198,199,200]. An antioxidant molecule, **resveratrol**, was used to invent multifunctional molecules by incorporation with **CQ** and **deferiprone** (**DFP**) [201,202]. **DFP** is a well-known Fe(II/III) chelator which is being examined for its potential to treat neurodegeneration. The information of clinical trials (phase II) is summarized in Table 1, and more information of **DFP** is discussed in Section 3.4 (vide infra).

## 3. Fe(II/III)

### 3.1. Fe(II/III) Distributions in the Nervous Systems

Fe is the most abundant metal in the brain [203]. Mostly, the oxidation states of Fe are ferrous [Fe(II)] and ferric [Fe(III)]. Due to the oxidation and reduction process between the ferrous and ferric states of Fe, it is called a redox-active metal ion, as with Cu(I/II). This redox-active property is important for the activity of various enzymes and proteins related to (i) O_2_ chemistry, (ii) electron transfer, (iii) gene regulation, and (iv) cell growth and differentiation [204,205,206,207]. To maintain the normal functions of the brain, Fe(II/III) are bound to the proteins in common, while labile Fe ions have been observed in intracellular pools with a concentration of up to 100 μM [208]. The concentrations of Fe(II/III) are different depending on the regions and cell types of the brain; 20–30 μM has been found in blood serum, and 0.5 to 1 mM has been found in neurons [209,210].

### 3.2. Homeostasis of Fe

Since the Fe ion is a redox-active metal ion, regulation of its level is essential to maintaining the normal biological functions [65]. The homeostasis of Fe(II/III) is tightly controlled by multiple proteins: heme proteins, transporters, Fe chaperones, ferrireductases, ferritin, transferrin (Tf), transferrin receptor 1 (TfR1), divalent metal transporter 1 (DMT1), and ferroportin (Fpn) [211]. Generally, in the human body, Fe(II/III) are bound to heme, ferritin, and Tf [212,213]. Two mechanisms of Fe(II/III) transportation have been reported: with and without Tf [214,215,216]. Non-Tf-bound Fe(II/III) would be delivered into the brain by astrocytes, oligodendrocytes, microglia, and/or albumin; however, heme- or ferritin-bound Fe(II/III) are not considered as non-Tf-bound Fe [217,218].

The major role of ferritin is storing Fe(III). It forms a 24 mer complex storing up to 4500 Fe(III)s composed of two subunits (chains): heavy (H) and light (L) chains [219,220]. These two chains present different functions. At a ferroxidase site in the H chain, Fe(II) is oxidized by coupling with O_2_. The ferroxidase site of human ferritin contains two Fe(II)s [221,222,223]. Glu27, Glu62, and His65, along with a water molecule, coordinate to Fe(II), and another Fe(II) is bound to Glu61, Glu62, and Glu107, with the assistance of Tyr34 and Gln141, in order to stabilize the structure [221,222,223]. The L chain promotes the nucleation of Fe core minerals such as ferrihydrite [224]. In addition to storing Fe ions, ferritin could reduce the generation of ROS to decrease oxidative stress, as well as lowering the toxicity induced by Fe(III) by inhibiting the interactions and reactions between Fe(III) and other biological molecules [219,224].

#### 3.2.1. Fe(II/III) Cross the BBB by Involvement of Tf

Most Fe ions are bound to Tf to be transported into the brain. Tf is a glycoprotein which is generated in the liver and has two Fe(II/III) binding sites at both the N- and C-terminal lobes composed of an aspartate, two Tyr, His residues (Asp63, Tyr95, Tyr188, and His249 on the N-terminal lobe; Asp392, Tyr426, Tyr517, and His585 on the C-terminal lobe), and a carbonate ion showing a high binding affinity towards Fe(III) (ca. *K*_a_ = 10^22^ M^−1^ at pH 7.4) [220,221,225,226]. About 34% of Tf has only one Fe(III) at the N-terminal lobe or C-terminal lobe, and 27% of Tf contains two Fe(III)s [226,227]. The suggested mechanism for the transportation of the Fe(III)-bound Tf complex through the BBB is receptor-mediated endocytosis [226,228,229]. At the membrane of brain capillary endothelial cells (BCEC), which maintain the integrity of the BBB, up to two Fe(III)–Tf complexes are captured by transferrin receptor-1 (TfR1) [226,228,230]. Then, Fe(III)–Tf tightly interacts with the receptor to be transported into endosomes, and the structural changes in Tf lead to opening the cleft of the Fe binding site to release the reduced form of Fe ions, Fe(II) [229]. Although the exact mechanism remains unclear, ferrireductases (e.g., STEAP3) have been proposed to be involved in this process [231].

Next, the released Fe(II) from Tf is bound to divalent metal transporter 1 (DMT1) to cross the endosomal membranes [232]. Fe(II) transport by DMT1 is an active and proton-dependent process; DMT1 could deliver Fe(II) the most effectively under mild acidic conditions [233,234]. Fe(II) from Tf could bind to Zrt-/Irt-like protein 8/14 (ZIP8/14) as well [235,236]. Although ZIP8 and ZIP14 are mostly involved in regulating Zn(II) levels, they have a significant role in the cellular uptake of Fe(II/III), with a poor understanding of the exact mechanism [237]. Unlike DMT1, ZIP8 and ZIP14 presented their optimal ability of Fe(II/III) transport under physiological pH [238,239].

The Fe ions are then exported to the brain by Fpn located on the cytoplasmic side of the plasma membrane [240]. For this process, extracellular ferroxidases are required [241]. Ceruloplasmin is one of the ferroxidases interacting with Fpn to enhance the incorporation of Fe ions into Tf, and efflux of the Fe ions from astrocytes [230,242].

#### 3.2.2. Fe(II/III) Transport without Tf

In CSF and interstitial fluids, labile Fe(II/III) could bind to other transporters such as albumin, lactoferrin, and p97 [243]. The lactoferrin receptor- and glycosylphosphatidylinositol-anchored p97-secreted pathways have also been reported to transport Fe(II/III) through the BBB [244,245]. Fe ions could bind to citrate and ascorbate as well. Most non-Tf-bound Fe(III) is bound to citrate in CSF, while the Fe(II/III)–ascorbate complex is observed with a low concentration (ca. nanomolar range). When Fe ions are not bound to Tf, known as non-Tf-bound Fe(II/III), the toxicity could be increased due to the high propensity to generate ROS [243].

Although numerous studies tried to reveal the exact mechanisms of Fe(II/III) transport across the BBB, they still remain unclear [246]. In order to understand the implications of Fe(II/III) in the brain, more details about Fe(II/III) miscompartmentalization, along with production of ROS in the pathogenesis of neurodegeneration and transport of Fe(II/III) without Tf, should be investigated.

### 3.3. Physiological and Pathological Functions of Fe in Nervous Systems

#### 3.3.1. Fe under Normal Conditions

Fe(II/III) are used for various enzymatic activities based on their common redox activity: mitochondrial respiration and O_2_ transport [247,248,249]. Specifically, Fe(II/III) help to generate energy in the form of ATP by the involvement of the electron transport chain as Fe(II/III)–S complexes and enzymatic cofactors of cytochromes. Since ATP synthesis requires O_2_, both hemoglobin and myoglobin containing Fe(II/III) are related to the process [250,251]. The generated energy is used for axonal and synaptic signaling in particular. For normally functioning mitochondria, the energy supplied by ATP is necessary [251].

In addition, Fe(II/III) are involved in the synthesis of myelin and neurotransmitters [247,248,249,252,253]. Myelin is a lipid-rich substance and required for fast information transmission (electronic signaling) from neurons to neurons by insulating nerve cell axons. For the myelination, the levels of Fe are important for the composition of myelin at the gestation and early post-natal periods [252,253]. In the process of myelin production, oligodendrocytes play important roles, with high concentrations of Fe(II/III) in the form of ferritin [254]. Cholesterol and lipids are two main components of myelin, and Fe(II/III) have been implicated to synthesize these biomolecules as a cofactor [254].

**Figure 3:**
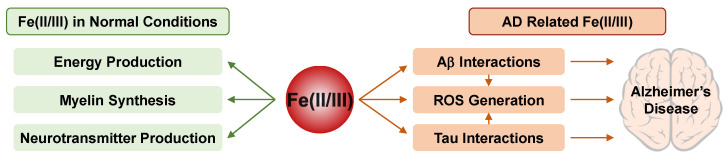
The roles and effects of Fe(II/III) in normal (left) and AD-affected (right) brains.

Additionally, Fe(II/III) are essential for the production of monoamine neurotransmitters, dopamine and serotonin, which regulate cognitive processes including emotion and arousal behaviors [255,256]. Dopaminergic systems, the tryptophan hydroxylases (necessary for serotonin synthesis), and glutamate dehydrogenase, along with γ-aminobutyric acid (GABA) transaminase (responsible for the synthesis and degradation of GABA), are affected by the levels of Fe(II/III) [257]. The basic roles of Fe(II/III) in the normal brain are summarized in Figure 3.

#### 3.3.2. Fe in Diseased Conditions

Under the conditions of AD, an abnormal concentration and distribution of Fe(II/III) have been reported. In particular, once the BBB is compromised due to aging and/or high levels of oxidative stress, the random transportation of Fe(II/III) across the BBB leads to miscompartmentalization of the ions in the brain [258]. Based on post-mortem analyses of AD-affected brains, accumulation of Fe(II/III) (up to 1 mM) was observed in amyloid plaques and neurofibrillary tangles, especially at the parietal cortex, putamen, and hippocampus, while the concentration of Fe(II/III) in the entire brain is similar to a healthy brain [259,260,261,262]. This dyshomeostasis of Fe(II/III) could lead to the malfunction of metalloenzymes and proteins in the brain which require Fe(II/III) as cofactors.

Moreover, both direct and indirect interactions between Fe(II/III) and Aβ/tau could be related to the onset and/or progression of AD, as shown in Figure 3 [263,264,265]. Fe(II/III) binding to Aβ could lead to the aggregation of proteins and toxicity induced by Aβ species. In addition, once Fe(II/III) are bound to Aβ, ROS would be generated by Fenton chemistry [264,265]. As Fe(II/III) themselves, without Aβ, could enhance the amounts of ROS, they could cause oxidative stress [266,267]. Indirectly, a high level of Fe(II/III) could decrease furin which increases the activity of β-secretase involving the amyloidogenic process of APP to generate Aβ [268]. Additionally, Fe(III) could directly bind to tau, causing its phosphorylation and aggregation of hyperphosphorylated tau [263]. Once tau is accumulated, the expression of heme oxygenase-1 would be increased; subsequently, more biliverdin, carbon monoxide, and labile Fe(II) catalyzed from heme would exist. These products from the catalysis of heme could enhance oxidative stress [268,269]. Although a recent study found that ceruloplasmin could reduce ferroptosis [270], these increased Fe(II/III) levels could lead to ferroptosis and damage the cellular antioxidant capacity in general [230,271]. Once the antioxidant ability has been compromised, ROS would accumulate, causing oxidative damage to nucleic acids, lipids, and proteins [230].

### 3.4. Regulators of Fe(II/III)

In order to reduce the risk of neurodegeneration by Fe(II/III), numerous studies tried to regulate the levels of metal ions, along with the antioxidant ability. Therefore, many chemicals have been invented as Fe(II) or Fe(III) chelators and examined for their ability to bind to Fe(II/III). In this section, we summarize the chemical agents capable of interacting with Fe(II/III).

The oxidation state of Fe should be considered to develop Fe chelators. Since Fe(III) is a hard acid based on the hard-soft acid and base (HSAB) principle, it prefers to bind to a hard base, such as an O donor atom. Fe(II), a borderline hard acid, prefers a borderline base, such as a N donor atom. Thus, various Fe(II/III) chelators have combinations of O and N as electron donor atoms [272,273,274].

**Figure 4:**
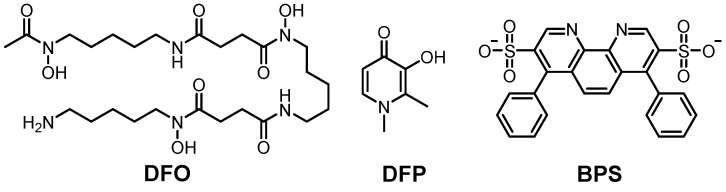
Representatives of Fe(II/III)-targeting molecules. Left: **deferoxamine** (**DFO**), *N*^1^-(5-aminopentyl)-*N*^1^-hydroxy-*N*^4^-(5-(N-hydroxy-4-((5-(N-hydroxyacetamido)pentyl)amino)-4-oxobutanamido)pentyl)succinamide; middle: **deferiprone** (**DFP**), 3-hydroxy-1,2-dimethylpyridin-4(1*H*)-one; right: **bathophenanthroline disulfonate** (**BPS**), 4,7-diphenyl-1,10-phenanthroline-3,8-disulfonate.

Among the chemical agents, **deferoxamine** (**DFO**) and **DFP** are the most well-known Fe(III) chelators (Figure 4) [272,273,275,276]. **DFO** is applied to Fe(II/III)-poisoning treatment by chelating metal ions (Figure 4). **DFO** forms the hexadentate Fe(III) coordination mode, forming an octahedral geometry [277]. Although **DFO** has limitations in cell permeability because of its hydrophilicity, it presented inhibition of Fe accumulation in rats and mice [275,278,279]. Moreover, **DFO** could improve the cognitive function of APP/PS1 transgenic mice as well as healthy mice [280,281]. It also presented an improvement in memory in an intracerebroventricular streptozotocin rat model assessed through the Morris water maze test (Table 3) [282]. **DFP** could interact with Fe(III) in a 1:3 metal-to-ligand ratio, forming an octahedral complex, and it has neuroprotective properties in neurons [273,276,283]. In addition, based on the structure of **DFP** with the benzothiazole moiety of **ThT**, **compound 2d** (from reference [284]) was developed, and it showed a strong Fe-chelating ability [284]. Moreover, recent animal studies showed that **DFP** could regulate the Aβ levels and phosphorylation of tau, leading to improved cognitive function in a transgenic mouse model of tauopathy, rTg4510 (Table 3) [285,286,287]. The results of clinical trials (phase II) of **DFP** are summarized in Table 1 [288]. Additionally, for chelating Fe(II), **bathophenanthroline disulfonate** (**BPS**; Figure 4; right) has been applied [289,290]. It could coordinate to Fe(II), forming an octahedral geometry, with a dissociation constant of 10^−17^ M [289,290]. Although **BPS** is water-soluble, due to the negative charges on sulfonate groups, it could not penetrate the cellular membrane [291].

**8-HQ** and its derivatives [e.g., **HLA20**, **GS(HQ)H**, **Compound 8g**, **VK28**, **M30**] have been developed, and they could interact with various metal ions [295,296,297,298]. **HLA20** could form a complex with Fe(III) at a 1:3 ratio, and its binding affinity to Fe(II) was 5.4 μM, determined by a fluorescence dequenching experiment of calcein [296]. **GS(HQ)H** was invented by a combination of **glutathione** and **8-HQ**, which could coordinate with Fe(III) and protect SHSY-5Y human neuroblastoma cells from H_2_O_2_- and 6-OHDA-induced damage [299]. **Compound 8g** (from reference [294]), designed by Knez and colleagues, also has Fe(II) binding ability [297]. **VK28** has been examined for its Fe(II/III)-chelating ability across the BBB [298]. **M30** containing an *N*-propargylamine group of **rasagiline** on the structure of **8-HQ** itself could bind Fe(II/III) directly with a 1:3 ratio and induce mRNA expression levels of the major antioxidant defense system composed of catalase, SOD-1, and glutathione peroxidase, in various brain regions [300]. Moreover, **M30** and **HLA20** significantly improved the cognitive deficits in a rat model [292,293,301]. In particular, **M30** could induce a neuroprotective effect and improve the memory of C57BL/J6 mice once they showed memory and behavioral impairment [294]. The information of animal studies performed with the treatment of Fe-targeting chemical agents is summarized in Table 3.

In addition to targeting only Fe(II) and/or Fe(III), multi-target molecules have emerged as a new strategy to investigate the pathology of AD and treat diseases. Multifunctional molecules are designed to simultaneously target multiple neurodegenerative pathological factors, which is anticipated to slow or even reverse the cognitive decline more effectively than single-target agents [1,302]. For targeting the risk factors of AD, the derivatives of **resveratrol**, **compound 3i** and **compound 4f** (from references [201,202]), were invented for interacting with Fe(III) as well as Fe(III)-bound Aβ_42_ [201,202]. These compounds could bind to Fe(III) with pFe(III) values of 20 and 19, respectively, along with having antioxidant activity in an ABTS assay. Moreover, they could affect the aggregation of Fe(III)–Aβ_42_ and disassemble preformed Fe(III)–Aβ_42_ aggregates [202]. Based on multifunctionality, a few chemical agents, although they have firstly been suggested as Fe(II/III) chelators, have recently been examined for their potentials as therapeutic agents for AD in various animals (Table 3) [280,281,282,285,286,287,292,293,294].

## 4. Amyloid Degrading Enzymes (ADE)

Although current strategies to treat AD could relieve symptoms temporarily, the disease cannot be cured completely. Therefore, in recent decades, inhibiting the generation of Aβ and modulating its aggregation pathways to form less neurotoxic aggregates have been intensively studied as potent methods to treat AD. In addition to these two aspects, clearance of amyloidogenic proteins from the brain is also important to have less toxic species in the brain. In Section 4 and Section 5, we provide a summary of (i) multiple ADEs, such as NEP, IDE, and ADAM10, along with their roles in the brain, and (ii) interrelations between redox-active metal ions and ADE affecting the activity and/or levels of ADE.

### 4.1. Neprilysin (NEP)

NEP is a zinc-dependent metalloprotease (type II integral membrane endopeptidase) which has been reported to be involved in the onset and/or progression of multiple diseases such as AD, heart failure, and diabetes [303,304,305,306]. Upon generation in Golgi, NEP exists in neutrophils, the kidney, the lungs, and the cerebral cortex in the brain [306,307,308]. It is mainly composed of α-helical structures with 749 amino acids in 3 domains: an N-terminal intracellular domain (27 amino acids), a transmembrane domain (23 amino acids), and an extracellular catalytic site (699 amino acids) [303,305,309]. For Zn(II) binding, 2N and 2O from His583, His587, Glu584, and Glu646 coordinate to Zn(II) with a tetrahedral geometry [310,311].

The S1, S1′, and S2′ subsites are binding pockets located near the Zn(II) binding site in the extracellular catalytic domain [303,305]. The pocket of the S1′ subsite specifically cleaves large hydrophobic and aromatic side chains at the hydrophobic amino acid residues, while the S2′ subsite could degrade bulky side chains [303,305]. NEP cleaves various vasoactive peptides (e.g., natriuretic peptides, bradykinin, adrenomedullin, angiotensin, substance P, enkephalins, endothelin, and Aβ) between the hydrophobic amino acids. Since Aβ has broad hydrophobic regions, it is an ideal substrate of NEP [15,312,313]. To have a high selectivity of substrates, the enzyme has a sterically hindered active site. The peptide bonds between Glu3 and Phe4, Gly9 and Tyr10, Phe19 and Phe20, and/or Ala30 and Ile31 of monomeric Aβ could be cleaved by NEP [314,315,316]. About 73% of monomeric Aβ_40_ and 27% of monomeric Aβ_42_ were degraded by NEP [317]. Oligomeric Aβ species could be degraded by NEP with a lower degree compared to monomeric Aβ, while it cannot cleave the amyloid precursor protein (APP) [312,317,318].

Based on the amyloid hypothesis, the accumulation of Aβ aggregates in the cerebral cortex and gray matter regions in the brain could cause AD. Therefore, the Aβ degrading activity and concentrations of NEP could affect the onset and/or progression of AD [319]. A lower level of NEP in CSF was shown in the early stage of AD which could progress to AD [320]. Its activity is varied based on pH. NEP presents the maximum cleavage activity at neutral pH against small peptides containing less than 50 amino acids [321]. The expression levels of NEP are not different between males and females [313].

In the mouse brain, higher Aβ levels were reported upon disruption of NEP expression [318]. Inactivation of NEP in a hAPP mouse model showed cognitive defects and impaired synaptic plasticity [318,322]. In addition, NEP in CSF has been suggested as a biochemical marker to monitor synaptic impairment since its activity was observed to be decreased by 12% in mild AD patients [323]. Furthermore, it has been reported that the increase in NEP levels could be a potent treatment for AD through Aβ metabolism as well as other mechanisms such as producing neuropeptide Y fragments [324,325,326,327]. Moreover, upon treatment of Tf-bound NEP, Wistar rats presented reduced levels of Aβ in both CSF and the brain [328]. Based on a recent meta-analysis of the expression and function of NEP in AD, it was reported that both the expression and activity of NEP were decreased in the cortex of elderly AD patients, supporting the idea that targeting NEP could be a potent strategy to treat and/or cure AD [329].

### 4.2. Insulin-Degrading Enzyme (IDE)

Another zinc-dependent metallopeptidase, IDE, which is a 113 kDa with 1019 amino acids, could cleave insulin and Aβ [330,331]. IDE is composed of two similar-sized domains: IDE-N and IDE-C. These two domains are connected by a loop containing 28 amino acids. A number of hydrogen bonds between IDE-N and IDE-C could continue to close the catalytic site located in IDE-N [332,333,334]. Since the active site is located in IDE-N, IDE-N itself could perform the proteolytic activity, while IDE-C could not show enzymatic activity [335]. Another difference between IDE-N and IDE-C is that the inner side of IDE-N is neutral or negatively charged, but IDE-C is positively charged. The positive charge of IDE-C could help the enzyme recognize the substrates [334]. The substrates of IDE contain β-structures such as Aβ [336,337,338]. At the catalytic site, the ^108^HXXEH^112^ motif is responsible for binding to Zn(II) [332]. In particular, Glu111 has an important role in the hydrolysis of substrates by acting as a base to the active catalytic water [332].

IDE is mostly located in the cytosol as well as mitochondria, peroxisomes, the plasma membrane, and CSF [339]. Therefore, IDE could control the levels of intracellular Aβ and reduce the damage induced by toxic Aβ species [340,341,342]. IDE could cleave the peptide bonds of Aβ between Val12 and His13, His13 and His14, His14 and Gln15, Val18 and Phe19, Phe19 and Phe20, Phe20 and Ala21, and/or Lys28 and Gly29 [333,343]. Additionally, IDE could act as a chaperone to interfere with the fibrillization of Aβ [344]. Furthermore, IDE has been reported as an important enzyme for the clearance of Aβ in hippocampal lysates, the cytoplasm, and cerebrospinal fluid [345,346]. Similar to NEP, IDE shows optimal cleavage activity at neutral pH as well [347].

In AD-affected brains, the levels of IDE were shown to increase, while the activity of IDE was reported to be decreased with aging, particularly in the early stage of the disease [347,348]. IDE and Aβ plaques were shown to be colocalized in the brain, indicating that IDE could be buried in the plaques and/or oxidized. Consequently, IDE could lose its amyloid-degrading ability, leading to a lower clearance of Aβ and higher aggregation of Aβ, resulting in neuronal damage [349,350]. Moreover, in vivo studies showed that IDE knockout animals have relatively high levels of Aβ, suggesting that the action of IDE to remove Aβ is important to control the amount of Aβ in the brain, and regulating the Aβ cleavage activity of IDE could be a potent strategy to treat AD [349,350].

### 4.3. ADAM10

The ADAM family, composed of zinc-dependent transmembrane metalloproteases, has been reported to cleave APP as well as cell adhesion and the proteolytic activity of signaling molecules and receptors [351,352,353,354]. Usually, ADAM contains ca. 750 amino acids as a signal peptide, a prodomain, a metalloprotease-like domain, a disintegrin-like domain, a Cys-rich domain, an EGF-like domain, a transmembrane domain, and cytoplamic tail [353]. The disintegrin-like domain acts as a ligand for integrin binding; however, this domain is not necessary for ADAM10 protease activity [355,356]. Among the ADAM family, ADAM9, ADAM10, and ADAM17 are involved in the generation of Aβ, and ADAM10 contributes the most to the proteolytic actions on APP [357,358].

ADAM10 is produced in the ER and matured and activated in Golgi by removing the prodomain. The size of matured ADAM10 is 68 kDa without activation (with the prodomain) [359,360]. While proADAM10 is located in Golgi, most of the activated ADAM10 is located on the plasma membrane [361]. ADAM10 exists as a dimeric form, and ^383^HEVGHNFGSPHD^344^ forms a Zn(II) binding site which contains the ^383^HEXXH^387^ motif. Three His residues play an important role in Zn(II) binding, and Gly between Phe and Ser constitutes a turn, whilst Glu acts as an acid/base catalyst [355,362]. Recently, the catalytic activity of ADAM10 has been revealed, and the activity of the enzymes could be regulated by a modulatory antibody. The C-terminal Cys-rich domain hinders the active site until there is direct binding of the proteolytic substrates [362,363,364,365].

APP, the target of ADAM10, undergoes a proteolytic cleavage reaction by β- and γ-secretases to generate Aβ [356,366]. ADAM10 acts as an α-secretase cleaving APP at different sites from β- and γ-secretases and produces non-toxic soluble APP (sAPPα), which has a neuroprotective function [355]. Therefore, upregulation of ADAM10 could be a promising strategy to treat AD by reducing toxic Aβ species and increasing neuroprotective sAPPα [355,367].

Furthermore, in neuronal systems, ADAM10 has a role in regulating synaptic proteins [368]. In particular, neuronal surface ADAM10 could be associated with AP2 for endocytosis. In AD patients, the level of ADAM–AP2 in the hippocampus was increased, while the activity of ADAM10 decreased upon interaction with AP2 in hippocampal neurons [368,369]. Moreover, in ADAM10 knockout mice, synaptic impairments, decreased neuromotor abilities, and reduced learning abilities were observed, indicating that the low level of ADAM10 could induce postsynaptic defects [370].

## 5. Redox-Active Metal Ions with ADE

Since both redox-active metal ions and ADE are related to the etiology of AD, investigation of their interactions along with enzymatic activity is necessary to further understand AD pathology. First, Cu ions could interact with NEP; in particular, the extracellular Cu(II) could reduce the levels of NEP in cells [371]. Similar results were obtained using a transgenic drosophila experiment that has a silent Ctr1 protein, along with a dissociation constant of NEP for Cu(II) determined as 1.04 (±0.07) μM. Even 100 μM of Zn(II) was not able to restore the activity of NEP [372,373]. Downregulation of NEP activity by Cu(II) was also observed in mouse neuroblastoma N2a cells. NEP activity was not inhibited at the transcription level. Rather, NEP turnover was blocked by specific proteasome inhibitors such as MG132 and lactacystin, suggesting a possible mechanism of NEP degradation would be the proteosome pathway [371]. Additionally, Fe could be related to decreased NEP activity. Once a chelator, **deferasirox**, chelates an Fe out from treated 18-month-old rats for 4 months, the A_β42_-degrading activity of NEP was recovered compared to the same aged rats [374].

Cu ions could downregulate the activity of IDE as well [372,375]. Inhibition of IDE activity by Cu(II) is reversible, and addition of Zn(II) restores IDE activity. However, inhibition of IDE activity by Cu(I) is irreversible [375]. Cu(I) binding to IDE blocks two Cys residues, Cys812 and Cys819, rendering the hydrophobic core destabilized. As a result, the Zn(II) binding site is closed, which is irreversibly inactivated [375]. Additionally, the proteolytic activity of IDE was inhibited by Cu(II); however, ubiquitin-activating (E1-like) activity was not affected up to 20 μM [376].

The interactions between Cu ions and ADAM10 have also been reported. There was no significant change in the ADAM10 level after 3-month Cu exposure of transgenic mice, while the ADAM10 level was dramatically increased after 9-month Cu exposure [377]. Unlike Cu ions, the overload of Fe ions could not significantly affect the expression levels of ADAM10, while the expression of ADAM17 increased. Additionally, Fe ions in the presence of peroxidative stress (treating with **Fenton**) could induce the expression of ADAM10 [378]. Moreover, Fe(II) could induce the mRNA levels of ADAM10 in cells along with the generation of sAPPα [379]. Recently, application of a Cu chelator, **tetrathiomolybdate** (shown in Figure 2; bottom row), to transgenic mice increased the levels of ADAM10, indicating that Cu chelators could activate the non-amyloidogenic processing of APP (less generation of Aβ) [380]. Nasal injection of a hydrogel containing another Cu-binding molecule, **D-penicillamine** (presented in Figure 2; bottom row), showed regulation of the ADAM10 level via the MT1/2 pathway, and enhanced lower production of Aβ in a transgenic mice experiment [163].

## 6. Conclusions

Redox-active metal ions, such as Cu(I/II) and Fe(II/III), are considered as major contributors to the complex chemical and biological reactions in the brain. In particular, Cu(I/II) and Fe(II/III) are important for oxygen consumption and the oxidative capacity of the neurons and glia in the brain. However, accumulation or miscompartmentalization of metal ions could be involved in the onset and/or progression of AD. Although various metal chelators have been developed for (i) regulating metal ions in the brain and (ii) investigating the involvement of metal ions in neurodegeneration, these have not been clearly revealed yet. Due to the complexity of the interrelationships between redox-active metal ions and other biological molecules (i.e., proteins, enzymes), multiple chemical agents have been examined as multifunctional molecules, reducing cytotoxicity associated with Cu(I/II), Fe(II/III), and Aβ. This strategy is promising; however, it is not the perfect way to treat the disease. Therefore, for treating AD, it is necessary to investigate and reveal the involvement of metalloenzymes, which can regulate the levels of Aβ in the brain such as ADE, in the pathogenesis of the disease, along with redox-active metal ions. In particular, Cu(I/II) and Fe(II/III) have potentials to interact with ADE and affect the enzymatic activity; however, their relationships still remain unclear. Current knowledge of the influence of Cu(I/II) and Fe(II/III) on the expression levels and activity of ADE is summarized in Table 4.

Through this review, we summarized (i) the roles, distributions, homeostasis, and transport of Cu(I/II) and Fe(II/III) in both healthy and AD-affected brains, (ii) chemicals designed for targeting metal ions, (iii) functions of multiple IDEs in the brain, and (iv) interrelationships between redox-active metal ions and ADE. Further understanding of the interrelationships between redox-active metal ions and ADE could lead to revealing the etiology of AD more clearly than before and developing successful treatments of the disease.

## Figures and Tables

**Table 1:** Clinical trials of chemical agents. **PBT-2** and **deferiprone** (**DFP**) have been examined their potential as treatments for neurodegeneration.

Chemical Agents	Clinical Trials (Periods)	Status
**PBT-2**	Phase 2 (December, 2006-December, 2007)	Completed (NCT00471211)
	Phase 2 (September, 2011-January, 2014)	Completed (ACTRN12611001008910)
	Phase 2 (July, 2013-January, 2015)	Completed (ACTRN12613000777796)
**Deferiprone (DFP)**	Phase 2 (October, 2016-September, 2019)	Completed (NCT02728843)
	Phase 2 (November, 2017-October, 2019)	Completed (ACTRN12617001578392)
	Phase 2 (January, 2018-December, 2021 (Estimated)]	Recruiting (NCT03234686)

**Table 2:** Recent animal studies conducted with Cu-targeting chemical agents. Results of in vivo experiments performed with **clioquinol** (**CQ**), **DP-109**, **DP-460**, **D-penicillamine**, and **tetrathiomolybdate** are summarized.

Chemical Agents	Animal Models	Outcomes	References
**Clioquinol (CQ)**	*C. elegans*	Enhancement of neuroprotective effect	[142]
**DP-109**	Transgenic mouse	Extension of life span and Reduction of amyloid plaques	[152,153]
**DP-460**	Transgenic mouse	Extension of life span and Reduction of amyloid plaques	[152,153]
	Rat	Detrimental effect on learning	[154]
**D-Penicillamine**	Transgenic mouse	Improvement of cognitive ability	[163]
**Tetrathiomolybdate**	Transgenic mouse	Decrease of the inflammatory cytokines	[167]

**Table 3:** Recent animal studies conducted with Fe-targeting chemical agents. Results of in vivo experiments performed with **deferoxamine** (**DFO**), **deferiprone** (**DFP**), **M30**, and **HLA20** are summarized.

Chemical Agents	Animal Model	Outcomes	References
**Deferoxamine (DFO)**	Transgenic mouse	Improvement of cognitive function	[280]
	Mouse	Improvement of memory	[281]
	Rat	Improvement of memory	[282]
**Deferiprone (DFP)**	Transgenic mouse	Improvement of cognitive function	[285,286,287]
**M30**	Rat	Recovery of memory impairment	[292,293]
	Mouse	Increase of neuroprotective effect Improvement of memory	[294]
**HLA20**	Rat	Recovery of memory impairment	[292,293]

**Table 4:** The reported relations between redox-active metal ions and ADE. The changes in expression levels and/or activity of NEP, IDE, and ADAM10 associated with Cu(I/II) and Fe(II/III).

		NEP	IDE	ADAM10
Cu(I/II)	Expression Levels	Decreased	Need to study	Increased
	Activity	Decreased	Decreased	Need to study
Fe(II/III)	Expression Levels	Need to study	Need to study	Increased
	Activity	Decreased	Need to study	Need to study
